# Loeffler's Syndrome Induced by the Transmigration of Strongyloides stercoralis to the Lungs: A Case Report and Literature Review

**DOI:** 10.7759/cureus.75897

**Published:** 2024-12-17

**Authors:** Bhupinder Singh, Sakshi Verma, Kanwal Rashid, Mason T Stoltzfus

**Affiliations:** 1 Internal Medicine, Icahn School of Medicine at Mount Sinai, Queens Hospital Center, New York, USA; 2 Medicine, Government Medical College, Amritsar, IND; 3 Neurosurgery, Penn State College of Medicine, Hershey, USA

**Keywords:** autoinfection, covid-19, ivermectin and albendazole, loeffler’s syndrome, parasitic infections, peripheral eosinophilia, strongyloides stercoralis, transmigration

## Abstract

Loeffler's syndrome is a rare, benign respiratory disease usually associated with peripheral eosinophilia, first described by Wilhelm Loeffler in 1932. It is caused by the larvae of helminths such as hookworms, *Ascaris*, and S*trongyloides* that transmigrate through the lungs during the active phase of infection. We present a case of a 53-year-old man who complained of a productive cough with intermittent hemoptysis and left-sided posterior chest pain. Initial evaluation revealed ground-glass opacity in the left upper lobe on chest X-ray and computed tomography (CT) scan, with laboratory results suggestive of leukocytosis, eosinophilia, and elevated IgE levels. After ruling out other potential diagnoses, strongyloidiasis was suspected. Serology for *Strongyloides stercoralis* was positive, and stool studies confirmed the presence of *Strongyloides* larvae. The patient was started on ivermectin and followed up in the outpatient clinic. Repeat chest CT after one month showed resolution of the previously noted left upper lobe infiltrate. This case report aims to describe this rare instance of Loeffler's syndrome and provide an update on the epidemiology, risk factors, and association of strongyloidiasis with other diseases and medications.

## Introduction

Loeffler's syndrome is a rare, nonmalignant respiratory disease usually associated with peripheral eosinophilia, first described by Wilhelm Loeffler in 1932 [1]. It is caused by larvae of helminths such as hookworms, *Ascaris*, and *Strongyloides* that transmigrate through the lungs during the active phase of infection. These larvae lodge in the lung bronchioles, triggering an eosinophilic inflammatory response that leads to pulmonary infiltrates [2]. Loeffler's syndrome is mainly characterized by pulmonary infiltrates, along with symptoms such as dry cough, wheezing, rales, and low-grade fever [3]. Peripheral eosinophilia is a key finding in Loeffler's syndrome. In 1952, pulmonary diseases with eosinophilia (either tissue or peripheral eosinophilia) were grouped into five major types, including simple pulmonary eosinophilia (Loeffler's syndrome), prolonged or recurrent pulmonary eosinophilia without asthma, pulmonary eosinophilia with asthma, tropical pulmonary eosinophilia, and polyarteritis nodosa [1]. The clinical presentation of Loeffler's syndrome is often mild and frequently asymptomatic, with spontaneous recovery occurring over a few days to months [4]. Radiological findings on chest X-ray may show round or oval opacities, ranging in size from a few millimeters to several centimeters, and may or may not be present in both lung fields [5]. However, in immunocompetent individuals, a low level of ongoing infection may persist for years, delaying clinical manifestations after the initial infection. 

## Case presentation

A 53-year-old man presented to the emergency department with a productive cough and intermittent hemoptysis for the past two months, associated with left-sided posterior chest pain occurring during coughing episodes and progressive shortness of breath for the last two weeks. He also reported three episodes of low-grade fever over the past two months, which resolved with acetaminophen. The patient is allergic to pollen and cat and dog dander. He has lived in the United States for the past 21 years and has not traveled recently. His medical history included chronic sinusitis, for which he underwent functional endoscopic sinus surgery (FESS), and a positive tuberculin test 11 years ago, although he denied any family history of active tuberculosis. His review of systems was negative for chills, rigors, night sweats, weight loss, new drug/environmental exposures, asthma, loss of consciousness, seizures, palpitations, syncope, nausea, vomiting, diarrhea, constipation, dysuria, recent travel, or sick contacts. 

Upon presentation, the patient had an oxygen saturation of 91% on room air, which improved to normal with 2 L of oxygen via nasal cannula. On examination, the patient was awake, alert, and oriented to time, place, and person. Vital signs included a respiratory rate of 18/min, pulse of 83/min, blood pressure of 121/79 mmHg, and temperature of 98.5 °F. Physical examination was negative for abnormalities in the head and neck, eyes, skin, and cardiovascular, gastrointestinal, genitourinary, musculoskeletal, neurological, and psychiatric/behavioral systems. The only positive finding was rhonchi in the left lung field on respiratory examination. 

**Figure 1:**
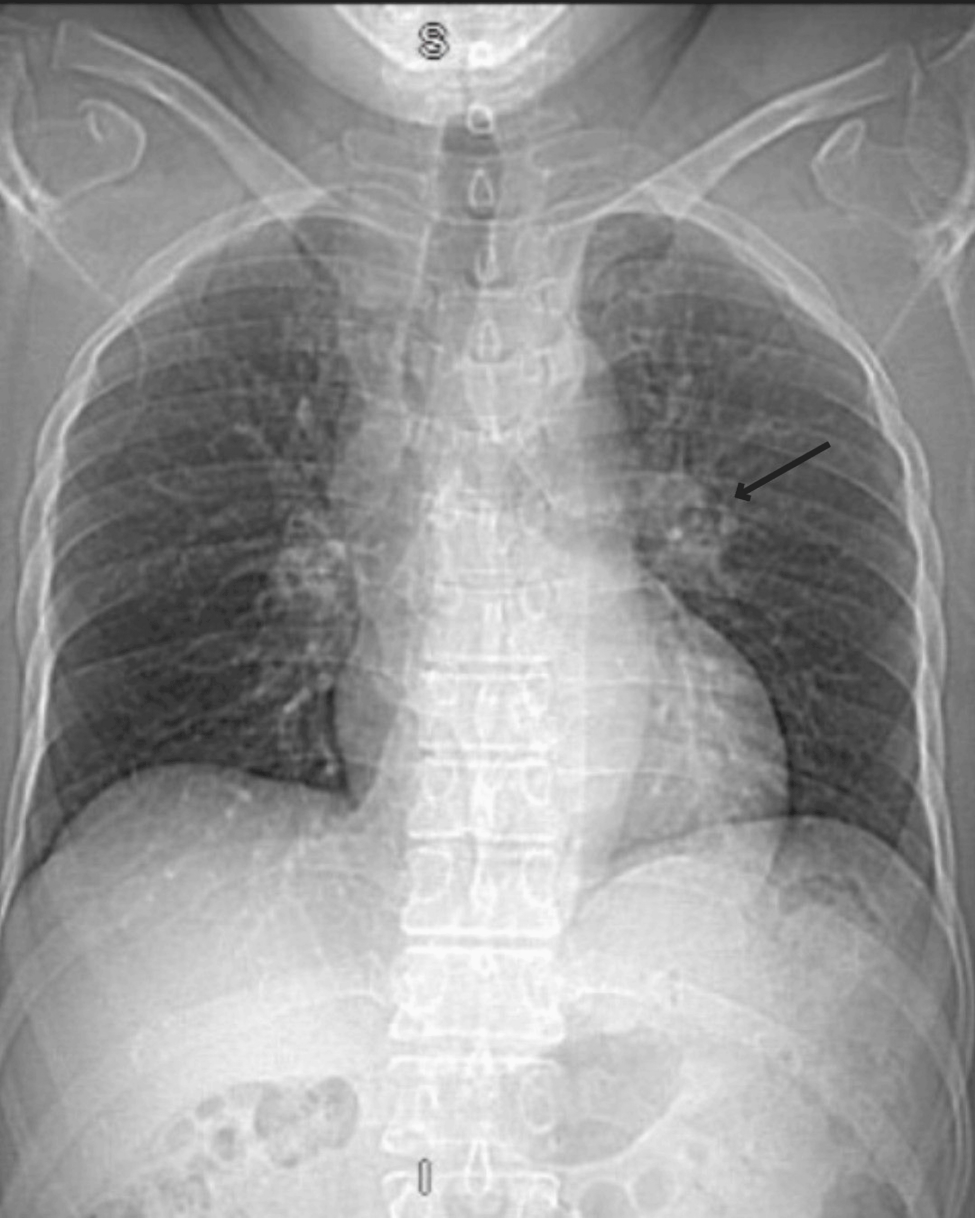
Chest X-ray showing ground-glass lesion around the left hilar region

**Figure 2:**
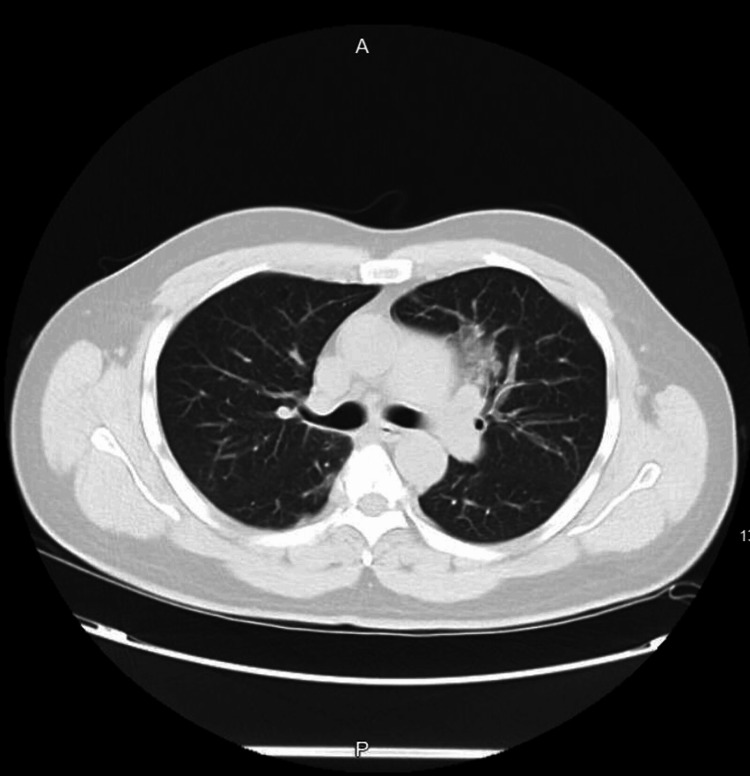
Chest CT showing ground-glass opacity in the anterior left upper lobe

The patient’s initial laboratory testing showed a white blood cell (WBC) count of 10.86 x 10^3^/mcL, eosinophils accounting for 19.5% of the WBC differential, and a venous lactate level of 3.5 mmol/L. The patient was tested for tuberculosis using the interferon-gamma release assay (IGRA), which was positive, but the chest X-ray and serial sputum cultures for acid-fast bacilli were negative. The chest X-ray showed hilar opacity in the left upper lung, and a subsequent chest CT revealed ground-glass opacity in the anterior left upper lobe, raising differential diagnoses including pneumonia, tuberculosis, fungal infection, and Loeffler's syndrome. To rule out these possibilities, the following tests were ordered: serum beta-D-glucan, urine legionella antigen, urine *Streptococcus pneumoniae* antigen, *Mycobacterium tuberculosis* (MTB) complex and rifampin (RIF) resistance PCR, *Mycoplasma* IgM antibody, hypersensitivity pneumonitis panel, antinuclear antibody (ANA) screen, and *Aspergillus galactomannan* antigen, all of which were negative. A *Strongyloides* antibody screen using enzyme-linked immunosorbent assay (ELISA) was performed within three days of presentation and returned positive. Serum IgE levels were elevated at 2538 KU/L. Due to a high suspicion of Loeffler's syndrome, serial stool cultures for ova and parasites were performed, which detected *Strongyloides stercoralis* larvae (Table 1, Figure 1, Figure 2).

**Table 1:** Laboratory tests RBC: red blood cell, Hct: hematocrit, MCV: mean corpuscular volume, TB: tuberculosis, PCR: polymerase chain reaction, HIV: human immunodeficiency virus, Ag/Ab: antigen/antibody, CMIA: chemiluminescent microparticle immunoassay, BUN: blood urea nitrogen, CRP: C-reactive protein, ANA: antinuclear antibody.

Parameter measures	Values	Reference range
White blood cell count	10.86	4.80-10.80 x 10^3^/mcL
Eosinophil	15.0	1.0%-4.0%
Eosinophil absolute	1.27	0.10-0.40 x 10^3^/mcL
Hemoglobin	14.9	14.0-18.0 g/dL
RBC	5.13	4.70-6.10 x 10^6^/mcL
Hct	44.9	42.0%-52.0%
MCV	87.5	80.0-90.0 fL
Platelet	232	150-450 x 10^3^/mcL
Neutrophil	38.9	44.0-70.0 %
Neutrophil absolute	3.92	2.10-7.60 x 10^3^/mcL
Lymphocytes	29.9	20.0%-45.0%
Lymphocyte absolute	3.01	1.00-4.90 x 10^3^/mcL
Basophil	1.5	0.0-2.0%
Basophil absolute	0.15	0.00-0.20 x 10^3^/mcL
Monocyte	6.9	2.0-10.0 %
Immature granulocyte absolute	0.03	0.00-0.20 x 10^3^/mcL
Mycoplasma IgM antibody	Negative	Negative
Serum beta-D-glucan	Negative	-
Aspergillus galactomannan antigen	Negative	-
QuantiFERON-Plus TB	Positive	-
Hypersensitivity pneumonitis panel	Negative	-
Procalcitonin	0.03	0.02-0.10 ng/mL
COVID-19 PCR	Negative	-
HIV 1 and 2 Ag/Ab by CMIA	Non-reactive	-
Allergy panel	Allergic to cat and dog dander, pollen peanuts, house dust mite	-
Sodium	144	136-145 mmol/L
Potassium	4.5	3.5-5.1 mmol/L
Chloride	106	98-108 mmol/L
CO_2_	28	22-29 mmol/L
BUN	11	6-23 mg/dL
Creatinine	0.81	0.70-1.20 mg/dL
Calcium	9.5	8.6-10.3 mg/dL
Phosphorus	2.2	2.5-4.5 mg/dL
Lactate	3.2	0.5-2.2 mmol/L
Serum IgE	2538	≤100 KU/L
*Strongyloides *antibodies	Positive	Positive
CRP	12.9	≤5.00 mg/L
ANA < 1:80	Negative	-
*Streptococcus pneumoniae* urine antigen	Negative	-
*Legionella *urine antigen	Negative	-

**Figure 3:**
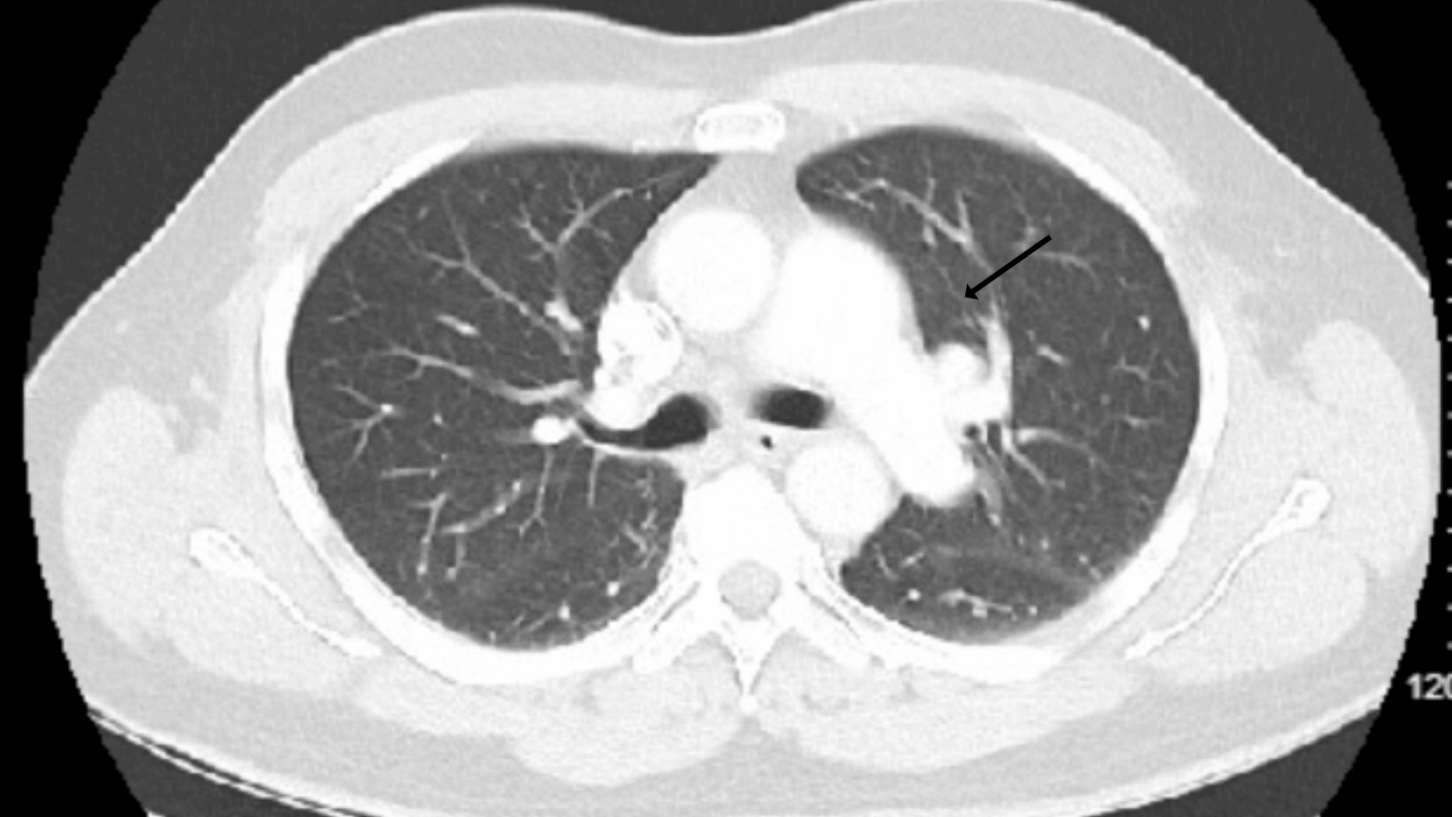
Chest CT showing resolution of the lesion after treatment

The patient was started on antibiotics for gram-negative coverage, including piperacillin and tazobactam 4.5 g every six hours, and ivermectin therapy at 200 mcg/kg once daily for two days. He remained hemodynamically stable and was discharged home. Follow-up was conducted four weeks later at the lung clinic. Repeat chest CT showed no evidence of focal consolidation or pleural effusion, and the previously noted left upper lobe infiltrate was no longer identified, confirming the resolution of the infiltrate (Figure 3).

## Discussion

Strongyloidiasis is a roundworm infection with an estimated global prevalence of up to 613.9 million cases (95% CI, 313.1-910.1), with Southeast Asia, Africa, and the Western Pacific accounting for 76.1% of cases [6]. In the United States, the infection is most common in the Southeastern states and Appalachian regions, where *S. stercoralis* larvae may be present in the soil [7]. Although hospitalization for strongyloidiasis is rare in the United States, it is associated with an increased risk of mortality. A population-based retrospective analysis using the National Inpatient Sample from 2003 to 2018 by Inagaki et al. identified 6,931 hospitalizations due to strongyloidiasis [8]. The highest infection rates were found in the Middle Atlantic division (47.1 cases per million population; adjusted odds ratio, 2.00; 95% CI, 1.58-2.53) and the East South Central division (27.5 cases per million; adjusted odds ratio, 2.77; 95% CI 2.02-3.80). Strongyloidiasis is more prevalent in areas with poor sanitation and among refugees, immigrants, and travelers [8]. 

*Strongyloides* can be transmitted through various modes, including direct skin contact with soil contaminated with human feces, fecal-oral transmission, and person-to-person transmission via fomites and sexual activity [9]. Although rare, nosocomial transmission has been described in several cases [10,11]. Once infected, the larvae mature into adults that penetrate the mucosa of the duodenum and jejunum, where they reproduce and lay eggs in the intestinal lumen. These eggs hatch, releasing larvae that may transform into filariform larvae, penetrating the gastrointestinal mucosa and leading to autoinfection. The larvae migrate via the lungs back to the intestines. Factors that may exacerbate autoinfection include constipation, diverticulosis, and the use immunosuppressive agents such as steroids, azathioprine, cyclophosphamide, antithymocyte globulin, doxorubicin, daunorubicin, ifosfamide, anti-CD3, chlorambucil, 6-mercaptopurine, methotrexate, bleomycin, adriamycin, melphalan, carmustine, and mitoxantrone, as well as total body irradiation [12]. In our case, the patient had a long-standing history of atopy and allergic rhinitis, and the use of corticosteroids could not be excluded. It is also important to note that immunosuppression increases the risk of disseminated disease due to unopposed accelerated autoinfection, even in cases with a remote history of initial infection [12]. Other conditions associated with impaired cell-mediated immunity, such as human T-lymphotropic virus type I (HTLV-I) infection, human immunodeficiency virus (HIV), hypogammaglobulinemia (including nephrotic syndrome and multiple myeloma), primary immune deficiencies, alcohol use disorder, and malnutrition, also increase the risk of disseminated *Strongyloides* infection. According to a meta-analysis by Ye et al., individuals with HTLV-1 are more likely to co-infect* S. stercoralis* in co-endemic areas (odds ratio, 3.2; 95% CI, 1.7-6.2) [13]. These individuals also have a higher chance of developing severe symptoms and a lack of response to therapy (odds ratio, 59.9; 95% CI, 18.1-198) [13]. Similarly, while disseminated infection can occur in individuals with advanced HIV, the risk is much lower compared to those with HTLV-1 infection [14]. Medications that cause immunosuppression, such as corticosteroids, cytotoxic drugs, and tumor necrosis factor inhibitors, may lead to hyperinfection syndrome, regardless of dose, duration, or route of administration. Case reports by Ghosh and Ghosh noted that even a short course (6-17 days) of corticosteroids (prednisolone 20-30 mg daily) could trigger hyperinfection syndrome, with a mortality rate of approximately 70% [15]. Case reports from Linoi Hospital’s intensive care unit described three immunocompetent patients who died from multiorgan failure and *S. stercoralis* septicemia following a short course of corticosteroid therapy (prednisolone 20-30 mg daily for 6-17 days) for peripheral eosinophilia, associated urticaria, angioneurotic edema, bronchospasm, and generalized aches and pains [15]. Recent studies by Rodríguez-Guardado et al. and Kim and Sivasubramanian have highlighted that the use of dexamethasone and other immunosuppressants (including azathioprine, cyclophosphamide, antithymocyte globulin, doxorubicin, daunorubicin, ifosfamide, anti-CD3, chlorambucil, 6-mercaptopurine, methotrexate, bleomycin, adriamycin, melphalan, carmustine, and mitoxantrone) for COVID-19 may also increase the risk of severe *S. stercoralis* infection [16,17]. 

Pulmonary strongyloidiasis (Loeffler's syndrome) may present initially with cough, throat irritation, dyspnea, and wheezing. However, symptoms secondary to inflammation induced by the transmigration of larvae, such as fever, hoarseness, choking, chest pain, hemoptysis, and palpitations, may also occur. Another important aspect of Loeffler's syndrome is that the transmigration of larvae can allow the entry of enteric organisms into the systemic circulation, leading to bacteremia and systemic sepsis. These bacteria may seed various sites, causing several extraintestinal manifestations such as pneumonia and meningitis [18]. Hence, systemic inflammation signs like fever and hemodynamic instability necessitate immediate evaluation for systemic bacterial infection through blood, sputum, and cerebrospinal fluid cultures. Chest X-ray and CT scan play an important role in assessing the morphology and location of pulmonary lesions, though findings can be nonspecific and suggest other etiologies. Hence, serology, stool studies, sputum, and bronchoalveolar lavage should be used to aid diagnosis [19]. 

In conclusion, due to the nonspecific nature of the symptoms, diagnosing Loeffler’s syndrome remains a challenge. Confirming the diagnosis requires a careful evaluation that combines pulmonary symptoms, peripheral blood eosinophilia, abnormal chest imaging, and histopathological abnormalities must be considered. After a thorough evaluation, the diagnosis should be established to rule out malignant conditions and differentiate it from other eosinophilic pulmonary disorders. Additionally, tracking the response to treatment is crucial to support the diagnosis [19] (Table 2).

**Table 2:** Analysis of different studies included in the discussion

Study	Type of study	Description
Buonfrate et al. [6]	Literature review	This study reported a global prevalence of *Strongyloides* as high as 613.9 million cases (95% CI, 313.1-910.1), with Southeast Asia, Africa, and the Western Pacific accounting for 76.1% of the cases.
Bradbury et al. [7]	Cross-sectional	*Strongyloides* infections occur among the inhabitants of the Southeastern states and Appalachian regions, where *S. stercoralis* larvae may be present in the soil.
Inagaki et al. [8]	Population-based retrospective analysis	Although hospitalizations due to strongyloidiasis are rare in the United States, they are associated with higher mortality rates and risk factors. Individuals who are impoverished or marginalized are more likely to be affected.
Vazquez et al. [9]	Case report	Individuals living with HIV who present with recurrent diarrhea and eosinophilia, regardless of their ethnicity or recent travel history, should be evaluated for parasitic infections, such as* S. stercoralis.*
Leapley et al. [10]	Letter	This letter described a *Strongyloides* outbreak investigation at a long-term care facility in Florida.
Jones et al. [11]	Case reports	These case reports described a *Strongyloides* investigation at a long-term care facility in Arizona.
Keiser and Nutman [12]	Literature review	Immunosuppressed individuals are at a higher risk of developing disseminated *Strongyloides* disease. In an immunocompromised state, the autoinfective cycle of *Strongyloides* can escalate into a potentially lethal hyperinfection syndrome. This condition is characterized by a high presence of infective filariform larvae in both the stool and sputum, along with clinical signs of increased parasite load and migration. These symptoms may include gastrointestinal bleeding and respiratory distress.
Ye et al. [13]	Meta-analysis	This study highlighted that the prevalence of *S. stercoralis* infection is higher in HTLV-1-positive individuals. Patients with co-infections are at a greater risk of developing severe symptoms and may not respond to treatment. Therefore, screening for both HTLV-1 and *Strongyloides* should be standard practice following a diagnosis of either condition.
Viney et al. [14]	Cross-sectional	This study found that disseminated *Strongyloides* infection can also occur in individuals with advanced HIV; however, the risk is much lower than compared to patients with HTLV-1 infection.
Ghosh and Ghosh [15]	Case reports	Corticosteroids can trigger hyperinfection syndrome, which carries a high mortality risk in patients with *Strongyloides* infection. These case reports support findings from several studies that describe the likely mechanism of *Strongyloides* dissemination during an immunosuppressed state.
Rodríguez-Guardado et al. [16]	Cross-sectional	The treatment of COVID-19 with dexamethasone and other immunosuppressants may increase the risk of severe *S. stercoralis *disease. A comprehensive national survey was carried out in Spain to gain a deeper insight into the diagnostic and therapeutic approach to strongyloidiasis in patients co-infected with SARS-CoV-2. A total of 189 responses were collected, with 121 (64%) advancing to the next stage of analysis. Among the surveyed centers, 84 (69.5%) did not implement a specific screening protocol for strongyloidiasis. Serological techniques were available in 42 centers (34.7%), while the remaining samples were sent to reference laboratories. Only 22 centers (18%) screened for strongyloidiasis in patients infected with SARS-CoV-2. The survey ultimately identified 227 cases of strongyloidiasis in individuals with SARS-CoV-2 infection, four of which progressed to severe hyperinfestation syndrome, resulting in one death.
Kim and Sivasubramanian [17]	Case report	This case report highlighted the need for *Strongyloides* screening in high-risk patients.
Ghoshal et al. [18]	Case reports	These case reports suggest that septicemia in patients with a fulminant *S. stercoralis* infestation may result from bacterial migration from the intestinal lumen.
Tran et al. [19]	Case report	This case report highlighted key criteria for diagnosing Loeffler’s syndrome. To confirm the diagnosis, a comprehensive evaluation of pulmonary symptoms, peripheral blood eosinophilia, abnormal chest imaging, and histopathological abnormalities is essential. After a thorough evaluation, the diagnosis should be established to rule out malignant conditions and differentiate it from other eosinophilic pulmonary disorders.

## Conclusions

Loeffler's syndrome is a rare pulmonary presentation of strongyloidiasis, often characterized by eosinophilia. The disease can persist for several days to months before clinical symptoms appear. Ivermectin (200 mcg/kg once daily for two days for immunocompetent individuals, and 200 mcg/kg daily for two days with a repeat regimen in two weeks for immunocompromised individuals) is effective in treating strongyloidiasis; however, more intensive regimens (200 mcg/kg daily until the symptoms resolve and stool or sputum specimens are negative for ≥2 weeks) may be required for more widespread disease. A disseminated disease is usually associated with immunodeficiency and the use of immunosuppressive agents.
